# Significance of Mucosa-Associated Microbiota and Its Impacts on Intestinal Health of Pigs Challenged with F18^+^ *E. coli*

**DOI:** 10.3390/pathogens11050589

**Published:** 2022-05-17

**Authors:** Marcos Elias Duarte, Sung Woo Kim

**Affiliations:** Department of Animal Science, North Carolina State University, Raleigh, NC 27695, USA; mduarte@ncsu.edu

**Keywords:** F18^+^ *Escherichia coli*, intestinal health, mucosa-associated microbiota, nursery pigs

## Abstract

The objective of this study was to evaluate the significance of jejunal mucosa-associated microbiota and its impacts on the intestinal health of pigs challenged with F18^+^ *Escherichia coli*. Forty-four newly-weaned pigs were allotted to two treatments in a randomized complete block design with sex as blocks. Pigs were fed common diets for 28 d. At d 7 post-weaning, pigs were orally inoculated with saline solution or F18^+^ *E. coli*. At d 21 post-challenge, feces and blood were collected and pigs were euthanized to collect jejunal tissue to evaluate microbiota and intestinal health parameters. The relative abundance of Firmicutes and Bacteroidetes was lower (*p* < 0.05) in jejunal mucosa than in feces, whereas Proteobacteria was greater (*p* < 0.05) in jejunal mucosa. F18^+^ *E. coli* increased (*p* < 0.05) protein carbonyl, *Helicobacteraceae*, *Pseudomonadaceae*, *Xanthomonadaceae*, and *Peptostreptococcaceae* and reduced (*p* < 0.05) villus height, *Enterobacteriaceae*, *Campylobacteraceae*, *Brachyspiraceae*, and *Caulobacteraceae* in jejunal mucosa, whereas it reduced (*p* < 0.05) *Spirochaetaceae* and *Oscillospiraceae* in feces. Collectively, jejunal mucosa-associated microbiota differed from those in feces. Compared with fecal microbiota, the change of mucosa-associated microbiota by F18^+^ *E. coli* was more prominent, and it was mainly correlated with increased protein carbonyl and reduced villus height in jejunal mucosa impairing the intestinal health of nursery pigs.

## 1. Introduction

The microbiota plays important roles on the maturation of the intestine and immune system and consequently affects the health of the host [1,2]. The physicochemical properties and the direct interaction with intestinal cells lead to a distinguishable composition of the microbiota along the gastrointestinal tract [3,4,5]. The microbiota in the intestinal lumen is more related to dietary compounds, whereas the mucosa-associated microbiota directly interacts with the mucus layer and the intestinal cells [6,7,8]. From the mucosa to the lumen the oxygen gradient is reduced which in turn makes the mucosal environment more propitious to aerobic and oxygen tolerant bacteria [3,9], whereas the mucus layer provides structures and substrates for bacteria with attachment characteristics and protein-degrading bacteria [10]. In addition, Zhao et al. [11] reported that the fecal microbiota is more similar to the microbiota in the lumen of the large intestine compared with the microbiota in the lumen of the small intestine. 

The mucosa-associated microbiota provides the first line of defense preventing the colonization of opportunistic pathogens [12,13]. Enterotoxigenic *Escherichia coli* can colonize the small intestinal mucosa facilitated by fimbrial or no-fimbrial adhesins and produce enterotoxins inducing luminal fluid secretion, and thus contributing to diarrhea [14,15]. The F18^+^ *E. coli* is highly prevalent in pigs causing post-weaning diarrhea (PWD) [14,16]. The PWD caused by enterotoxigenic *E. coli* infection is well known to induce microbiota dysbiosis [17,18,19]. During infection, the increased nitrate and oxygen concentration may increase the proliferation of aerobic and facultative anaerobic bacteria, and reduce anaerobic bacteria [18,20,21]. The F18^+^ *E. coli* challenge has been associated to increased inflammatory response and oxidative stress, consequently affecting intestinal morphology and enterocyte proliferation [18,22,23,24]. According to Duarte at al. [18], F18^+^ *E. coli* challenge markedly affects the jejunal mucosa-associated microbiota in pigs 14 days post-challenge, although the symptoms of PWD caused by F18^+^ *E. coli* generally last up to 11 days post-challenge [14,18,22,25]. It has been demonstrated that dietary intervention has a longer-lasting effect on mucosa-associated microbiota rather than on fecal microbiota in nursery pigs [4,26]. Furthermore, Liu et al. [27] reported that understanding the modulation of mucosa-associated can be a key factor to provide a more precise nutritional intervention to enhance intestinal health.

Therefore, it was hypothesized that the jejunal mucosa-associated microbiota is directly related to intestinal health of nursery pigs and the impacts of F18^+^ *E. coli* challenged to nursery pigs are greater to jejunal mucosa-associated microbiota than in fecal microbiota. To test the hypothesis, the objective of this study was to evaluate the significance of jejunal mucosa-associated microbiota and its impacts on the intestinal health of pigs challenged with F18^+^ *E. coli*.

## 2. Results

### 2.1. Growth Performance and Fecal Score

**Figure 1:**
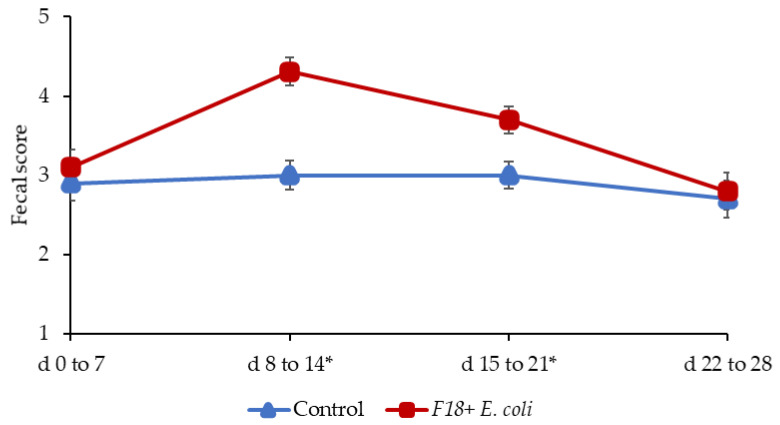
Fecal score of pigs challenged with enterotoxigenic F18^+^ *E. coli* on d 7 post-weaning. * d 8 to 14: *p* < 0.05; * d 15 to 21: *p* < 0.05.

Pigs challenged with F18^+^ *E. coli* showed a reduced growth performance at the end of experiments. During the pre-challenge period (d 0 to 7) the fecal scores of pigs was not different (Figure 1), confirming that pigs were in normal fecal score before the F18^+^ *E. coli* challenge. The F18^+^ *E. coli* challenge increased (*p* < 0.05) the fecal score of pigs from d 8 to 14 and from d 15 to 21 post-weaning. The F18^+^ *E. coli* challenge did not affect the fecal score of pigs from d 22 to 28 post-weaning.

### 2.2. Alpha Diversity of Microbiota

**Figure 2:**
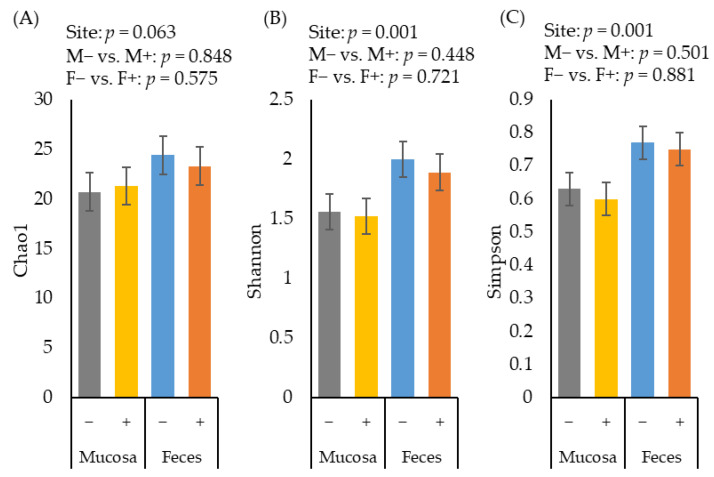
Alpha diversity of fecal and jejunal mucosa-associated microbiota estimated with Chao1 richness (**A**), Shannon diversity (**B**), and Simpson diversity (**C**) in pigs at d 21 after challenge with enterotoxigenic *E. coli* F18^+^. Site: mucosa and feces. F18^+^ *E. coli* challenge: no challenge: (−) and challenge: (+). Mucosa vs. feces: effect of site on microbiota; M− vs. M+: effect of F18^+^ *E. coli* on jejunal mucosa-associated microbiota; F− vs. F+: effect of F18^+^ *E. coli* on fecal microbiota.

The alpha diversity of fecal microbiota estimated with Chao1 tended to be greater (*p* = 0.063) than the diversity of jejunal mucosa-associated microbiota (Figure 2A). The alpha diversity of fecal microbiota estimated with Shannon (Figure 2B) and Simpson (Figure 2C) were greater (*p* < 0.05) than the diversity of jejunal mucosa-associated microbiota. The F18^+^ *E. coli* challenge did not affect the alpha diversity of fecal or jejunal mucosa-associated microbiota at d 21 post-challenge.

### 2.3. Beta Diversity of Microbiota

**Figure 3:**
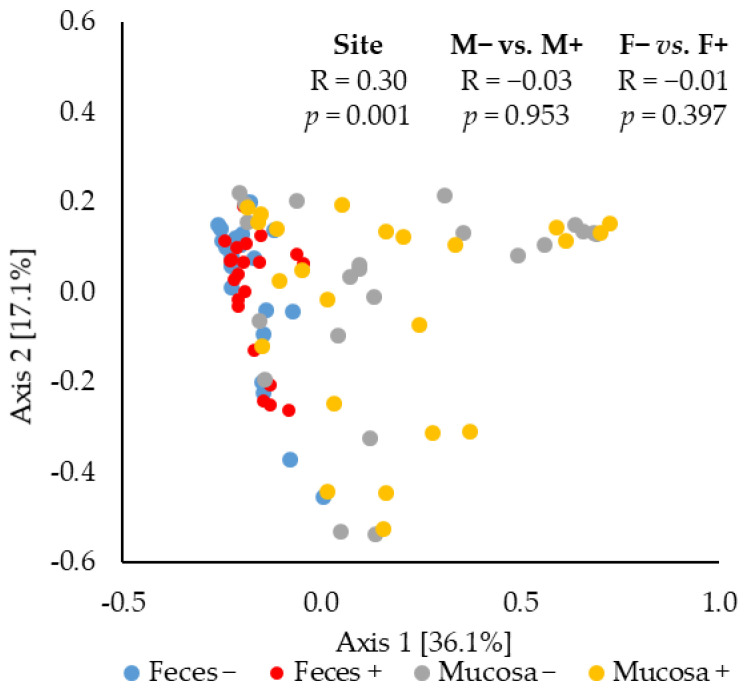
Beta diversity of fecal and jejunal mucosa-associated microbiota in nursery pigs challenged with enterotoxigenic F18^+^ *E. coli*. Principal coordinates analysis (PCoA) plot based on Bray–Curtis distance showed distinct clusters in the mucosa-associated microbiota (orange) and fecal microbiota (blue). The analysis of similarity (ANOSIM) procedure was used for the significance of the clustering pattern between jejunal mucosa-associated and fecal microbiota. Site: mucosa and feces. F18^+^ *E. coli* challenge: no challenge: (−) and challenge: (+). Mucosa vs. feces: effect of site on microbiota; M− vs. M+: effect of F18^+^ *E. coli* on jejunal mucosa-associated microbiota; F− vs. F+: effect of F18^+^ *E. coli* on fecal microbiota.

The principal coordinates analysis (PCoA) based on Bray–Curtis distance showed that the jejunal mucosa-associated microbiota was different (ANOSIM, mucosa-associated microbiota vs. fecal microbiota: R = 0.30, *p* < 0.05) from the fecal microbiota (Figure 3). The F18^+^ *E. coli* challenge did not affect the beta diversity of fecal or jejunal mucosa-associated microbiota at d 21 post-challenge.

### 2.4. Relative Abundance of Microbiota 

Firmicutes, Proteobacteria, and Bacteroidetes were the most abundant phylum, accounting for 94 and 95% of all microbiota in mucosa and feces, respectively (Table 1). Proteobacteria was the most abundant phylum in the jejunal mucosa of pigs followed by Firmicutes, and Bacteroidetes. In feces, Firmicutes was the most abundant, followed by Bacteroidetes and Proteobacteria. The relative abundance of Firmicutes and Bacteroidetes was lower (*p* < 0.05) in jejunal mucosa than in feces. The relative abundance of Proteobacteria was greater (*p* < 0.05) in jejunal mucosa than in feces. The F18^+^ *E. coli* challenge did not affect the relative abundance of fecal and jejunal mucosa-associated microbiota at phylum level.

The relative abundance of *Prevotellaceae, Veillonellaceae, Lachnospiraceae, Succinivibrionaceae, Ruminococcaceae, Acidaminococcaceae, Porphyromonadaceae, Erysipelotrichaceae, Eubacteriaceae, Cytophagaceae, Spirochaetaceae*, and *Rikenellaceae* was lower (*p* < 0.05) in jejunal mucosa than in feces (Table 2). The relative abundance of *Helicobacteraceae, Campylobacteraceae, Pseudomonadaceae, Enterobacteriaceae, Moraxellaceae, Pseudanabaenaceae*, and *Caulobacteraceae* was greater (*p* < 0.05) in jejunal mucosa than in feces. The relative abundance of *Lactobacillaceae, Clostridiaceae, Streptococcaceae, Brachyspiraceae, Nostocaceae, Bacillaceae, Bifidobacteriaceae, Eubacteriaceae, Hyphomicrobiaceae, Bacteroidaceae, Flavobacteriaceae*, and *Mycoplasmataceae* in jejunal mucosa was not different from feces.

The relative abundance of *Helicobacteraceae, Pseudomonadaceae, Xanthomonadaceae*, and *Peptostreptococcaceae* was greater (*p* < 0.05) in jejunal mucosa of nursery pigs challenged with F18^+^ *E. coli*. The relative abundance of *Campylobacteraceae, Enterobacteriaceae, Brachyspiraceae*, and *Caulobacteraceae* was lower (*p* < 0.05) in jejunal mucosa of nursery pigs challenged with F18^+^ *E. coli*. The relative abundance of Bacillaceae tended to be lower (*p* = 0.089) in jejunal mucosa of nursery pigs challenged with F18^+^ *E. coli*. The relative abundance of *Spirochaetaceae, Oscillospiraceae*, and Others was lower (*p* < 0.05) in feces of nursery pigs challenged with F18^+^ *E. coli*. The relative abundance of *Acidaminococcaceae* tended to be greater (*p* = 0.088) in feces of nursery pigs challenged with F18^+^ *E. coli*.

The relative abundance of *Prevotella, Succinivibrio, Megasphaera, Faecalibacterium Phascolarctobacterium, Selenomonas, Dialister, Roseburia, Acidaminococcus, Eubacterium, Oscillibacter, Herbaspirillum, Tepidimonas*, and *Ruminococcus* was lower (*p* < 0.05) in jejunal mucosa than in feces (Table 3). The relative abundance of *Helicobacter, Pseudomonas, Chlamydia, Acinetobacter*, and *Tepidimonas* was greater (*p* < 0.05) in jejunal mucosa than in feces. The relative abundance of *Campylobacter* (*p* = 0.071), *Chlamydia* (*p* = 0.056), and *Massilia* (*p* = 0.051) tended to be greater in jejunal mucosa than in feces. The relative abundance of *Lactobacillus, Clostridium, Mitsuokella, Streptococcus, Bifidobacterium, Gemmiger, Bacillus, Coprococcus, Catenibacterium, Anaerovibrio, Dorea, Brachyspira, Enterococcus*, and *Treponemain* in jejunal mucosa was not different from feces.

The relative abundance of *Helicobacter* and *Tepidimonas* was greater (*p* < 0.05) in jejunal mucosa of nursery pigs challenged with F18^+^ *E. coli*. The relative abundance of *Campylobacter* was lower (*p* < 0.05) in jejunal mucosa of nursery pigs challenged with F18^+^ *E. coli*. The relative abundance of *Pseudomonas* (*p* = 0.073) and *Bacillus* (*p* = 0.084) tended to be lower (*p* < 0.05) in jejunal mucosa of nursery pigs challenged with F18^+^ *E. coli*. The relative abundance of *Dialister* and *Acidaminococcus* was greater (*p* < 0.05) in feces of nursery pigs challenged with F18^+^ *E. coli*. The relative abundance of *Oscillibacter* and Tepidimonas was lower (*p* < 0.05) in feces of nursery pigs challenged with F18^+^ *E. coli*. 

The relative abundance of *Prevotella copri, Prevotella sp., Succinivibrio dextrinosolvens, Faecalibacterium prausnitzii, Phascolarctobacterium succinatutenswas, Dialister succinatiphilus, Roseburia faecis, Selenomonas lipolytica, Acidaminococcus fermentans, Selenomonas bovis, Selenomonas bovis*, and *Treponema porcinum* was lower (*p* < 0.05) in jejunal mucosa than in feces (Table 4). The relative abundance of *Prevotella stercorea* (*p* = 0.068), *Lactobacillus salivarius* (*p* = 0.072), and *Campylobacter lanienae* (*p* = 0.057) tended to be lower in jejunal mucosa than in feces. The relative abundance of *Helicobacter mastomyrinus, Helicobacter rappini, Acinetobacter radioresistens*, and *Acinetobacter lwoffii* was greater (*p* < 0.05) in jejunal mucosa than in feces. The relative abundance of *Lactobacillus mucosae* (*p* = 0.052), *Streptococcus alactolyticus* (*p* = 0.080), *Chlamydia suis* (*p* = 0.058), *Streptococcus infantarius* (*p* = 0.062), and Others (*p* = 0.055) tended to be greater in jejunal mucosa than in feces.

The relative abundance of *Helicobacter rappini* was greater (*p* < 0.05) in jejunal mucosa of nursery pigs challenged with F18^+^ *E. coli*. The relative abundance of *Campylobacter coli* was lower (*p* < 0.05) in jejunal mucosa of nursery pigs challenged with F18^+^ *E. coli*. The relative abundance of *Acinetobacter lwoffii* was lower (*p* < 0.05) in jejunal mucosa of nursery pigs challenged with F18^+^ *E. coli*. The relative abundance of *Prevotella copri* tended to be lower (*p* = 0.073) in jejunal mucosa of nursery pigs challenged with F18^+^ *E. coli*. The relative abundance of *Helicobacter mastomyrinus* (*p* = 0.079), *Helicobacter equorum* (*p* = 0.054), and *Streptococcus hyointestinalis* (*p* = 0.058) tended to be greater in jejunal mucosa of nursery pigs challenged with F18^+^ *E. coli*. The relative abundance of *Dialister succinatiphilus, Acidaminococcus fermentans*, and *Prevotella* sp. were greater (*p* < 0.05) in feces of nursery pigs challenged with F18^+^ *E. coli*. The relative abundance of *Selenomonas lipolytica, Campylobacter lanienae*, and *Treponema porcinum* was lower (*p* < 0.05) in feces of nursery pigs challenged with F18^+^ *E. coli*. 

The F18^+^ *E. coli* challenge did not affect the concentration of TNF-α and protein carbonyl in serum of nursery pigs at d 21 post-challenge (Table 5). However, at d 21 post-challenge, the concentration of protein carbonyl was greater (*p* < 0.05) in jejunal mucosa of nursery pigs challenged with F18^+^ *E. coli*. 

The villus height was reduced (*p* < 0.05) in nursery pigs challenged with F18^+^ *E. coli* (Table 6). The VH:CD ratio was increased (*p* < 0.05) in nursery pigs challenged with F18^+^ *E. coli*. The F18^+^ *E. coli* challenge did not affect the jejunal crypt depth and the proliferation of enterocytes in crypts. 

The concentration of TNF-α in serum was positively correlated with *Veillonellaceae* (r = 0.32; *p* < 0.05), *Acidaminococcaceae* (r = 0.36; *p* < 0.05), *Porphyromonadaceae* (r = 0.43; *p* < 0.05), *Eubacteriaceae* (r = 0.33; *p* < 0.05), *Cytophagaceae* (r = 0.33; *p* < 0.05), *Pseudanabaenaceae* (r = 0.35; *p* < 0.05), *Oscillospiraceae* (r = 0.37; *p* < 0.05), *Spiroplasmataceae* (r = 0.33; *p* < 0.05), *Lactobacillus mucosae* (r = 0.51; *p* < 0.05), *Megasphaera hominis* (r = 0.49; *p* < 0.05), and *Campylobacter lanienae* (r = 0.49; *p* < 0.05) in jejunal mucosa (Table 7). The concentration of TNF-α in jejunal mucosa was negatively correlated with *Bifidobacteriaceae* (r = −0.63; *p* < 0.05) in jejunal mucosa. The concentration of TNF-α in jejunal mucosa of nursery pigs was positively correlated with *Mitsuokella jalaludinii* (r = 0.45; *p* < 0.05), *Dialister succinatiphilus* (r = 0.52; *p* < 0.05), *Mitsuokella multacida* (r = 0.42; *p* < 0.05), and *Acidaminococcus fermentans* (r = 0.60; *p* < 0.05) in jejunal mucosa. The concentration of protein carbonyl in serum was positively correlated with *Oscillospiraceae* (r = 0.42; *p* < 0.05), *Lactobacillus johnsonii* (r = 0.49; *p* < 0.05), *Spiroplasmataceae* (r = 0.69; *p* < 0.05), *Megasphaera hominis* (r = 0.45; *p* < 0.05), and *Campylobacter lanienae* (r = 0.66; *p* < 0.05) in jejunal mucosa. The concentration of protein carbonyl in jejunal mucosa was positively correlated with *Erysipelotrichaceae* (r = 0.32; *p* < 0.05), *Spiroplasmataceae* (r = 0.44; *p* < 0.05), *Acidaminococcus fermentans* (r = 0.30; *p* < 0.05), and *Acinetobacter lwoffii* (r = 0.33; *p* < 0.05) in jejunal mucosa. The concentration of protein carbonyl in jejunal mucosa was negatively correlated with *Bifidobacteriaceae* (r = −0.33; *p* < 0.05) in jejunal mucosa. The villus height was positively correlated with *Veillonellaceae* (r = 0.33; *p* < 0.05), *Brachyspiraceae* (r = 0.34; *p* < 0.05), *Pseudomonadaceae* (r = 0.33; *p* < 0.05), *Oscillospiraceae* (r = 0.42; *p* < 0.05), *Campylobacter coli* (r = 0.33; *p* < 0.05), and *Megasphaera hominis* (r = 0.37; *p* < 0.05) in jejunal mucosa. The crypt depth was positively correlated *Lactobacillaceae* (r = 0.41, 0.006), *Pseudanabaenaceae* (r = 0.36; *p* < 0.05), *Helicobacter rappini* (r = 0.32; *p* < 0.05), *Dialister succinatiphilus* (r = 0.30; *p* < 0.05), *Megasphaera hominis* (r = 0.41; *p* < 0.05), *Selenomonas bovis* (r = 0.33; *p* < 0.05), and *Campylobacter lanienae* (r = 0.35; *p* < 0.05) in jejunal mucosa. The crypt depth was negatively correlated with *Prevotellaceae* (r = −0.36; *p* < 0.05), *Succinivibrio dextrinosolvens* (r = −0.50, 0.001), *Succinivibrionaceae* (r = −0.39; *p* < 0.05), *Faecalibacterium prausnitzii* (r = −0.55; *p* < 0.05), Lachnospiraceae (r = −0.40; *p* < 0.05), *Ruminococcaceae* (r = −0.36; *p* < 0.05), *Roseburia faecis* (r = −0.38, 0.013), Clostridiaceae (r = −0.49; *p* < 0.05), *Gemmiger formicilis* (r = −0.38; *p* < 0.05), *Pseudomonadaceae* (r = −0.30; *p* < 0.05), *Xanthomonadaceae* (r = −0.32; *p* < 0.05), *Mycoplasmataceae* (r = −0.34; *p* < 0.05), and *Dorea longicatena* (r = −0.40; *p* < 0.05) in jejunal mucosa. The villus height to crypt depth ratio was positively correlated with *Lachnospiraceae* (r = 0.31; *p* < 0.05), *Clostridiaceae* (r = 0.48; *p* < 0.05), *Xanthomonadaceae* (r = 0.32; *p* < 0.05), *Succinivibrio dextrinosolvens* (r = 0.35; *p* < 0.05), *Helicobacter rappini* (r = −0.34; *p* < 0.05), *Faecalibacterium prausnitzii* (r = 0.43; *p* < 0.05), *Roseburia faecis* (r = 0.34; *p* < 0.05), *Acinetobacter lwoffii* (r = 0.33; *p* < 0.05), and *Dorea longicatena* (r = 0.35; *p* < 0.05) in jejunal mucosa. The villus height to crypt depth ratio was negatively correlated with *Lactobacillaceae* (r = −0.32; *p* < 0.05) and *Helicobacter rappini* (r = −0.34; *p* < 0.05) in jejunal mucosa.

The concentration of TNF-α in serum was positively correlated with *Veillonellaceae* (r = 0.37; *p* < 0.05), *Streptococcaceae* (r = 0.47; *p* < 0.05), *Acidaminococcaceae* (r = 0.31; *p* < 0.05), *Eubacteriaceae* (r = 0.34; *p* < 0.05), *Cytophagaceae* (r = 0.41; *p* < 0.05), *Flavobacteriaceae* (r = 0.38; *p* < 0.05), *Prevotella copri* (r = 0.49; *p* < 0.05), *Phascolarctobacterium succinatutens* (r = 0.36; *p* < 0.05), *Streptococcus alactolyticus* (r = 0.36; *p* < 0.05), *Megasphaera hominis* (r = 0.46; *p* < 0.05), *Streptococcus hyointestinalis* (r = 0.34; *p* < 0.05), *Streptococcus infantarius* (r = 0.37; *p* < 0.05), and *Dorea longicatena* (r = 0.30; *p* < 0.05) in feces (Table 8). The concentration of TNF-α in serum was negatively correlated with *Oxalobacteraceae* (r = −0.31; *p* < 0.05), *Succinivibrio dextrinosolvens* (r = −0.49; *p* < 0.05), *Selenomonas lipolytica* (r = −0.30; *p* < 0.05), and *Treponema porcinum* (r = −0.32; *p* < 0.05) in feces. The concentration of protein carbonyl was positively correlated with *Acidaminococcaceae* (r = 0.31; *p* < 0.05) and *Prevotella copri* (r = 0.35; *p* < 0.05) in feces. The villus height was positively correlated with *Flavobacteriaceae* (r = 0.33; *p* < 0.05), *Dialister succinatiphilus* (r = 0.41; *p* < 0.05), *Lactobacillus salivarius* (r = 0.34; *p* < 0.05), and *Mitsuokella multacida* (r = 0.34; *p* < 0.05), whereas it was negatively correlated with *Treponema porcinum* (r = −0.30; *p* < 0.05) in feces. The crypt depth was positively correlated with *Acidaminococcaceae* (r = 0.48; *p* < 0.05), *Cytophagaceae* (r = 0.34; *p* < 0.05), *Flavobacteriaceae* (r = 0.35; *p* < 0.05), *Oscillospiraceae* (r = 0.32; *p* < 0.05), *Prevotella copri* (r = 0.47; *p* < 0.05), *Prevotella stercorea* (r = 0.31; *p* < 0.05), *Prevotella* sp. (r = 0.45; *p* < 0.05), *Faecalibacterium prausnitzii* (r = 0.34; *p* < 0.05), and *Dialister succinatiphilus* (r = 0.42; *p* < 0.05) in feces. The crypt depth was negatively correlated with *Oxalobacteraceae* (r = −0.33; *p* < 0.05) *Bifidobacteriaceae* (r = −0.32; *p* < 0.05), *Succinivibrio dextrinosolvens* (r = −0.42; *p* < 0.05), *Lactobacillus kitasatonis* (r = −0.33; *p* < 0.05), *Lactobacillus johnsonii* (r = −0.37; *p* < 0.05), *Selenomonas lipolytica* (r = −0.45; *p* < 0.05), and *Selenomonas bovis* (−0.41; *p* < 0.05) in feces. The villus height and crypt depth ratio was positively correlated with *Lactobacillaceae* (r = 0.32; *p* < 0.05), *Succinivibrionaceae* (r = 0.32; *p* < 0.05), *Selenomonas lipolytica* (r = 0.39; *p* < 0.05), and *Selenomonas bovis* (r = 0.46; *p* < 0.05) in feces. The villus height and crypt depth ratio was negatively correlated with *Ruminococcaceae* (r = −0.33; *p* < 0.05), *Acidaminococcaceae* (r = −0.30; *p* < 0.05), *Oscillospiraceae* (r = −0.31; *p* < 0.05), and *Prevotella* sp. (r = −0.42; *p* < 0.05) in feces. The enterocyte proliferation in crypt was positively correlated with *Ruminococcaceae* (r = 0.48; *p* < 0.05) and *Phascolarctobacterium succinatutens* (r = 0.41; *p* < 0.05) in feces. The enterocyte proliferation in crypt was positively correlated with *Oxalobacteraceae* (r = −0.52; *p* < 0.05), *Lactobacillus mucosae* (r = −0.60; *p* < 0.05), and *Lactobacillus delbrueckii* (r = −0.51; *p* < 0.05) in feces.

## 3. Discussion

The different physicochemical properties, the substrate availability, and the interaction with the immune system along the gastrointestinal tract play important roles in the modulation of intestinal microbiota [2,11,28]. The microbiota in the intestinal lumen is more related to dietary compounds, whereas the mucosa-associated microbiota directly interacts with the mucus layer and the intestinal cells [6,7,8]. In addition, the motility and the ability to attach to the intestinal mucosa are also determinants for intestinal colonization and consequently differentiate the mucosa-associated microbiota from the fecal microbiota [29,30]. 

In this study, the similarity index of jejunal mucosa-associated microbiota was only 0.30 when compared with the fecal microbiota. Considering that the similarity of microbiota from the ileal lumen was 0.85 when compared with ileal mucosa-associated microbiota [28], the result in the current study is in accordance with Zhao et al. [11] who reported that the similarity of the luminal digesta in the ileum was 0.38 when compared with fecal microbiota. In addition, the jejunal mucosa-associated microbiota showed a lower alpha diversity when compared with the fecal microbiota. The jejunum is the major site for digestion and absorption of energy, amino acids, and fat [31]. The differences in the oxygen gradient, pH, and nutrient availability in the jejunal mucosa and feces are the main factors altering the composition of microbiota [2,4]. 

The differences between the fecal and the jejunal mucosa-associated microbiota were mainly due to the changes in the relative abundance of Proteobacteria, Firmicutes, and Bacteroidetes observed in this study. The oxygen gradient decreases from mucosa to the lumen and from the small intestine to the large intestine [9,32]. In addition, the luminal content in the jejunum is different from those in the large intestine. Beyond the digestive functions, the small intestine contains more immune cells than the large intestine [33] which may exert more interaction between the immune system and the microbiota in the small intestine [34]. It has been reported that the mucosa-associated microbiota can directly crosstalk with immune cells regulating the immune response [6,7,35]. In this study, the abundance of *Helicobacteraceae*, *Campylobacteraceae*, *Pseudomonadaceae*, *Enterobacteriaceae Xanthomonadaceae*, and *Moraxellaceae* was greater in jejunal mucosa, whereas *Prevotellaceae*, *Veillonellaceae*, *Lachnospiraceae*, *Succinivibrionaceae*, *Ruminococcaceae*, *Acidaminococcaceae*, *Erysipelotrichaceae*, *Eubacteriaceae*, and *Spiroplasmataceae* were greater in feces. Kelly et al. [36] also reported similar differences in microbiota composition in jejunal mucosa and the luminal content in the cecum. *Campylobacter* and *Helicobacter* are microaerophilic bacteria with great motility and attaching ability [29,30] which benefits their colonization in the mucosa of the small intestine. *Prevotellaceae*, *Veillonellaceae*, *Lachnospiraceae*, *Ruminococcaceae*, *Erysipelotrichaceae*, and *Eubacteriaceae* have been associated with feces or luminal content of the large intestine of pigs [37,38]. Interestingly, the abundance of *Lactobacillaceae* and *Clostridiaceae* did not differ between jejunal mucosa and feces. *Lactobacillus* and *Clostridium* are fiber-degrading bacteria that produce short-chain fatty acids (SCFAs), which further would affect the intestinal health of the host [39,40].

The mucosa-associated microbiota provides the first line of defense preventing the growth of opportunistic pathogens [12,13]. The commensal *Helicobacteraceae*, *Campylobacteraceae*, *Pseudomonadaceae*, *Enterobacteriaceae*, and *Clostridiaceae* are considered opportunistic pathogens that potentially stimulate the immune system conferring a protecting role on the intestinal barrier [1,2,41]. However, in events such as weaning stress, the balance of mucosa-associated microbiota can be disrupted increasing the susceptibility to overgrowth of these pathogens resulting in enteric diseases [2]. 

This study evaluated the effect of F18^+^ *E. coli* on the modulation of microbiota and its correlation with the health of pigs. Previous studies have demonstrated that post-weaning diarrhea caused by F18^+^ *E. coli* disrupts the microbiota composition [17,18]. According to Duarte at al. [18], F18^+^ *E. coli* challenge markedly affects the jejunal mucosa-associated microbiota in pigs 14 days post-challenge. In the current study, 21 days post-challenge, the F18^+^ *E. coli* did not affect the microbial diversity; however, it increased *Helicobateraceae*, *Pseudomonadaceae*, *Xanthomonadaceae*, and *Peptostreptococcaceae*, whereas it competitively reduced *Campylobacteraceae*, *Clostridiaceae*, *Enterobacteriaceae*, *Brachyspiraceae*, and *Caulobacteraceae*, in jejunal mucosa. In feces, the F18^+^ *E. coli* challenge reduced *Spirochaetaceae* and *Oscillospiraceae.* Bacteria can directly promote the immune response in the intestine due to the cell wall structures and secreted compounds [42,43,44]. The fimbrial or no-fimbrial adhesins facilitate the adhesion of *E. coli* to the mucosa [14,15]. The adherence of the F18^+^ *E. coli* and the secretion of enterotoxins cause an imbalance in the fluid to the intestinal lumen [44,45], which can increase the inflammatory response and the oxidative stress resulting in damaged intestinal morphology and increased enterocyte proliferation [18,22,25]. The disrupted microbiota can further increase the inflammatory response, increasing the nitrate and oxygen concentration which in turn increases the proliferation of aerobic and facultative anaerobic bacteria and reduce anaerobic bacteria [18,20,21]. Additionally, the mucosa-associated microbiota has been reported to be more susceptible to changes in the small intestine [26,46]. These results suggested that the F18^+^ *E. coli* has a long-lasting effect on the intestinal health of pigs, although diarrhea caused by *E. coli* generally lasts 7 to 11 days [14,18,22,25].

The increased abundance of Proteobacteria, including *Helicobacter* and *Pseudomonas*, can reduce the mucous layer [47], increasing the immune response [18]. The increased immune response can increase the production of oxygen reactive subspecies, consequently inducing oxidative stress in jejunal mucosa, as reported in this study. The reduced mucus layer and the increased oxidative stress can lead to enterocyte apoptosis reducing the villus height [48,49]. Proteobacteria species have been previously associated with inflammatory and oxidative stress markers and damaged villi [1,18,50]. In this study *Campylobacter lanienae* and *Helicobacter rappini* were positively correlated with TNF-α, protein carbonyl, and crypt depth, whereas they were negatively correlated with the VH:CD. *Prevotellaceae*, *Bifidobacteriaceae*, and *Faecalibacterium prausnitzii* were negatively correlated with TNF-α, protein carbonyl, and crypt depth. The crypt depth and the VH:CD are indicators of enterocyte proliferation and villus damage. Greater crypt depth indicated greater enterocyte proliferation in crypts, whereas a greater VH:CD indicates lower enterocyte proliferation in crypts due to the reduced villus damage [51,52]. 

In conclusion, the jejunal mucosa-associated microbiota had greater abundance of Proteobacteria and lower abundance of Firmicutes and Bacteroidetes compared with fecal microbiota. F18^+^ *E. coli* affected the jejunal mucosa-associated microbiota by increasing *Helicobacteraceae*, *Pseudomonadaceae*, *Xanthomonadaceae*, and *Peptostreptococcaceae*, competitively reducing *Campylobacteraceae*, *Enterobacteriaceae*, *Brachyspiraceae*, and *Caulobacteraceathe.* The changes in jejunal mucosa-associated microbiota were correlated with the increased oxidative stress and reduced villus height caused by the F18^+^ *E. coli* challenge. Compared with fecal microbiota, jejunal mucosa-associated microbiota was effectively correlated with key intestinal health parameters indicating the importance of mucosa-associate microbiota in the jejunum as biomarkers to potentially predict the deleterious effects of F18^+^ *E. coli* infection.

## 4. Materials and Methods

The experimental protocols used in this study were reviewed and approved by the Institutional Animal Care and Use Committee at North Carolina State University following the North Carolina State Animal Care and Use Procedures (REG 10.10.01).

### 4.1. Animals, Diets, Experimental Design, and Inoculum

Forty-four newly weaned pigs (22 barrows and 22 gilts) at 21 d of age from three experiments were used in this study. The initial BW of pigs were 4.9 ± 0.1, 6.3 ± 0.1, and 6.2 ± 0.5 for Exp. 1, 2, and 3, respectively. The experiments were conducted at the Metabolism Education Unit at North Carolina State University. Within each experiment, pigs were allotted to two treatments (no challenge and Challenge) in a randomized complete block design with sex and initial BW as blocks. All pigs were fed a common basal diet meeting the nutritional requirements suggested by NRC [53] for 28 d divided in 2 phases (Table 9). 

At d 7, pigs were inoculated with an oral inoculation of saline solution or F18^+^ *E. coli* at 4 × 10^9^ CFU for pigs in Exp. 1 and 5.2 × 10^9^ CFU for pigs in Exp. 2 and 3. All pigs were purchased from a commercial farm in North Carolina, USA and were not vaccinated against *E. coli.*

The F18^+^ *E. coli* inoculum was prepared using the strain F18ac (O147) producing heat-stable toxin A (STa) and heat-stable toxin B (STb), following our standard protocol as previously described by Duarte et al. [18]. The F18ac (O147) was selected based on its stronger capacity to adhere to the small intestinal receptors in weaned pigs [24,54] causing diarrhea [18,22]. The oral inoculation was divided into two doses.

### 4.2. Sample Collection and Processing

Fresh fecal samples and blood were collected from all pigs at d 21 post-challenge. Blood samples were collected from the jugular vein of all pigs at d 21 of the experiment. The blood samples (7 mL) were collected into vacutainers without anticoagulant (BD, Franklin Lakes, NJ, USA) and centrifuged at 3000× *g* for 15 min at room temperature. The serum was aliquoted and stored at −80 °C for further analysis to measure the concentration of tumor necrosis factor α (TNF-α), as an indicator of inflammatory response and protein carbonyl, as an indicator of oxidative stress status. 

At d 21, all pigs were euthanized to collect jejunal samples. Tissues from mid-jejunum (3 m after the duodenojejunal junction) were collected, rinsed with a 0.9% saline solution, and fixed in 10% formalin. The jejunal tissue was used for histological measurements and to measure the enterocyte proliferation in crypts. The enterocyte proliferation rate was analyzed by immunohistochemistry using Ki67 staining. A section of mid-jejunum (10 cm) was longitudinally opened, rinsed with a 0.9% saline solution, and scraped to obtain mucosa samples. The samples were placed into 2 mL tubes, snap-freezing in liquid nitrogen, and stored at −80 °C for further analyses to evaluate the microbiota composition and measure the concentration of total protein, TNF-α, and protein carbonyl. Prior to the analysis the samples (0.5 g) were suspended in 1 mL of phosphate-buffered saline (PBS) and homogenized on ice using a tissue homogenizer (Tissuemiser; Thermo Fisher Scientific Inc. Waltham, MA USA). The homogenized mucosa samples were centrifuged at 14,000× *g* for 15 min. The supernatant was aliquoted in 4 aliquots and stored at −80 °C until analysis to measure the concentration of total protein, TNF-α, and protein carbonyl.

### 4.3. Inflammatory and Oxidative Stress Parameters

The concentrations of total protein, TNF-α, and protein carbonyl were measured by colorimetric methods using commercial kits as previously described by Cheng et al. [1]. The absorbance was measured using a plate reader (Synergy HT, BioTek Instruments, Winooski, VT, USA) and the software (Gen5 Data Analysis Software, BioTek Instruments). The concentrations were obtained by the standard curves generated from the concentration and absorbance of the standard from each parameter. The concentration of TNF-α and protein carbonyl were measured in serum and mucosa as indicators of inflammatory and oxidative stress status, respectively. Total protein concentration was measured to normalize the concentration of TNF-α and protein carbonyl in the mucosa.

Total protein concentration was measured using the bicinchoninic acid (BCA) Protein Assay (23225#, Thermo Fisher Scientific Inc.). Serum and mucosa samples were diluted (1:80 and 1:50, respectively) in PBS to meet the working range of 20 to 2000 µL. The absorbance was measured at 562 nm. The concentration of TNF-α was measured using the porcine TNF-α ELISA Kit (PTA00, R&D Systems, Inc., Minneapolis, MN, USA) with the working range at 23 to 1500 pg/mL, and the absorbance measured at 450 and 540 nm as previously described by Sun et al. [22]. The concentration of protein carbonyl in serum and jejunal mucosa was measured using the OxiSelect Protein carbonyl ELISA Kit (Cell Biolabs, Inc., San Diego, CA, USA). Prior to the analysis, the samples were diluted with PBS to reach the concentration of protein at 10 µg/mL. The working range was 0.0 to 7.5 nmol/mg of protein, and the absorbance was measured at 450 nm as previously described by Cheng et al. [1].

### 4.4. Jejunal Morphology and Crypt Cell Proliferation

**Figure 4:**
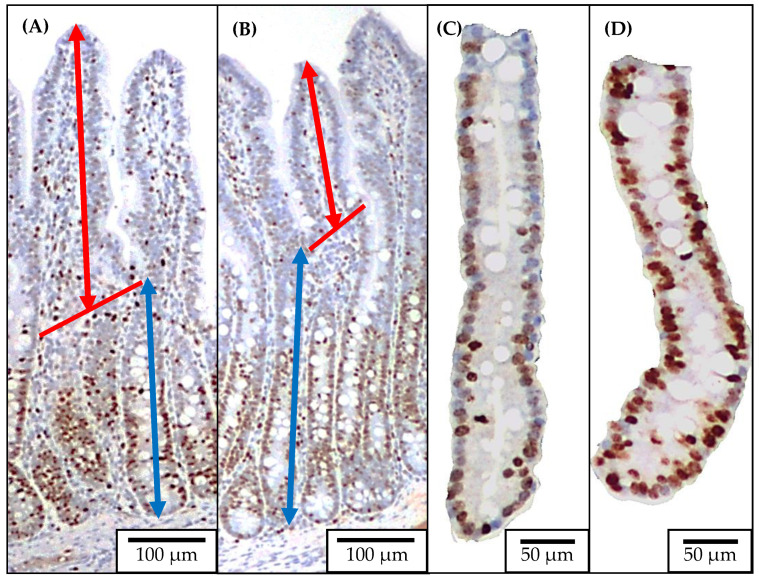
Representative images of the immunohistochemistry (Ki67) staining for jejunal morphology and crypt cell proliferation. Ten images at 40× of well-oriented villi and their associated crypts ((**A**): no challenged; (**B**): F18^+^ *E. coli* challenged) were obtained for measuring villus height (from the top to the base of villus as indicated with double arrow line in red) and crypt depth (from the base of villus to the bottom of the crypt as indicated with double arrow line in blue). Ten images at 100× of the crypts ((**C**): no challenged; (**D**): F18^+^ *E. coli* challenged) were obtained for measuring the percentage of positive Ki67 staining cells.

Two sections of jejunal tissue were placed into a cassette in 70% ethanol after 48 h in 10% formalin and sent to the North Carolina State University Histology Laboratory (College of Veterinary Medicine, Raleigh, NC) for Ki67^+^ staining as previously described by Sun et al. [22] (Figure 4). The jejunal morphology was evaluated by measuring the villus height, and crypt depth using a microscope Olympus CX31 with camera Infinity 2-2 digital CCD. Pictures of well-shaped villi and crypts were taken in 40× magnification to measure the villus height and crypt depth. The villus height to crypt depth ratio (VH:CD) was further calculated. Pictures of jejunal crypts were taken in 100× magnification to measure the enterocyte proliferation rate by measuring the Ki67^+^ cells. The percentage of Ki67^+^ cells in the total cells in the crypt was evaluated using the ImageJS software, and an indicator of the enterocyte proliferation in the crypt was used [55].

### 4.5. Relative Abundance and Diversity of the Fecal and Mucosa-Associated Microbiota in Jejunum

Jejunal mucosa and fecal samples were used for DNA extraction using the QIAamp DNA Stool Mini Kit (51504) following the manufacturer instructions. The extracted DNA samples were sent to the MAKO Medical Laboratories in Raleigh, North Carolina for qPCR analysis of 16S rDNA sequences as previously described by [50]. The Ion Chef instrument was used for the preparation of samples for template and the Ion S5 system was used for sequencing. The Ion 16S Metagenomics Kit (A26216, Thermo Fisher Scientific Inc.) was used to amplify the variable regions V2, V3, V4, V6, V7, V8, and V9 of the 16S rRNA gene. The Ion Xpress Plus Fragment Library Kit (Cat. 4471269, Thermo Fisher Scientific Inc.) was used to prepare the libraries from the amplified regions and the Ion Code Barcode Adapters 1-384 Kit (A29751, Thermo Fisher Scientific Inc.) was used for barcoding and multiplexing of the prepared libraries. The Ion Universal Library Quantitation Kit (A26217, Thermo Fisher Scientific Inc.) was used to quantify the libraries. The Torrent Suite Software (version 5.2.2) was used to process the sequences producing unaligned bam files for further analysis. The Ion Reporter Software Suite (version 5.2) of bioinformatics analysis tools was used for sequence data analysis, alignment to GreenGenes and MicroSeq databases, alpha and beta diversity plot generation, and OTU table generation. All samples had a depth of sequencing coverage greater than 1000×. The OTU data were transformed to relative abundance for further statistical analysis, and the OTU data with less than 0.05% abundance within each level were combined as “others”.

### 4.6. Statistical Analysis

Data were analyzed using the Mixed procedure of SAS Software. The sites (jejunal mucosa and feces) and the F18^+^ *E. coli* challenge (no challenge: (M− and F−) and challenge: (M+ and F+)) were the fixed effects. The experiments were considered random effects. The LSMEANS statement was used to calculate the least squared mean. To evaluate the microbiota data, pre-planned orthogonal contrasts were made to test the effect of the site (jejunal mucosa vs. feces) on microbiota, the effect F18^+^ *E. coli* on jejunal mucosa-associated microbiota (M− vs. M+), and the effect F18^+^ *E. coli* on fecal microbiota (F− vs. F+). The procedure CORR was used to test the correlation among microbiota (fecal and jejunal mucosa-associated) and inflammatory, oxidative stress, and morphological measurements. Statistical differences were considered significant with *p* < 0.05 and tendency with 0.05 < *p* < 0.10.

## Figures and Tables

**Table 1:** Relative abundance of fecal and jejunal mucosa-associated microbiota at the phylum level in pigs at d 21 after challenge with enterotoxigenic *E. coli* F18^+^.

Site ^1^	Mucosa		Feces			*p* Value ^3^		
Challenge ^2^	−	+	−	+	SEM	Site	M− vs. M+	F− vs. F+
Firmicutes	36.11	35.07	48.61	46.73	5.40	0.014	0.878	0.781
Proteobacteria	35.93	43.47	12.27	15.10	5.60	<0.001	0.287	0.689
Bacteroidetes	21.55	16.04	35.23	32.15	3.24	<0.001	0.230	0.500
Chlamydiae	3.72	2.91	<0.01	1.71	2.05	0.203	0.769	0.523
Spirochaetes	1.53	0.11	1.09	0.34	0.64	0.867	0.122	0.410
Actinobacteria	0.41	0.57	1.17	0.55	0.36	0.316	0.754	0.231
Cyanobacteria	0.33	1.51	0.03	0.03	0.74	0.223	0.254	0.996
Others	0.31	0.21	1.21	3.10	1.57	0.357	0.987	0.167

^1^ Site: mucosa and feces. ^2^ F18^+^ *E. coli* challenge: no challenge: (−) and challenge: (+). ^3^ Mucosa vs. feces: effect of site on microbiota; M− vs. M+: effect of F18^+^ *E. coli* on jejunal mucosa-associated microbiota; F− vs. F+: effect of F18^+^ *E. coli* on fecal microbiota.

**Table 2:** Relative abundance of fecal and jejunal mucosa-associated microbiota at the family level in pigs at d 21 after challenge with enterotoxigenic *E. coli* F18^+^.

Site ^1^	Mucosa		Feces			*p* Value ^3^		
Challenge ^2^	−	+	−	+	SEM	Site	M− vs. M+	F− vs. F+
*Helicobacteraceae*	14.31	29.89	0.62	0.77	5.21	<0.001	0.017	0.982
*Prevotellaceae*	19.83	14.77	29.39	28.81	2.92	<0.001	0.219	0.888
*Lactobacillaceae*	13.37	14.34	9.37	11.62	3.44	0.331	0.843	0.645
*Veillonellaceae*	8.27	7.16	18.95	17.14	2.96	<0.001	0.713	0.550
*Campylobacteraceae*	8.30	2.04	1.29	0.41	1.98	0.039	0.027	0.757
*Pseudomonadaceae*	1.80	4.74	<0.01	<0.01	1.60	<0.001	0.034	0.951
*Clostridiaceae*	4.47	2.27	1.60	1.67	1.87	0.235	0.288	0.975
*Enterobacteriaceae*	5.24	1.44	0.12	2.14	1.65	0.038	0.021	0.140
*Streptococcaceae*	2.96	4.31	1.31	1.36	2.16	0.138	0.535	0.982
*Chlamydiaceae*	3.56	2.27	0.02	<0.01	1.90	0.065	0.587	0.979
*Lachnospiraceae*	1.31	1.94	4.81	4.73	0.84	<0.001	0.541	0.942
*Succinivibrionaceae*	0.79	0.46	7.48	10.00	3.68	<0.001	0.917	0.428
*Ruminococcaceae*	1.25	1.34	5.64	4.86	0.64	<0.001	0.908	0.319
*Brachyspiraceae*	2.44	0.08	0.55	0.03	0.78	0.214	0.035	0.634
*Moraxellaceae*	1.13	0.84	<0.01	0.03	0.52	0.029	0.667	0.980
*Oxalobacteraceae*	0.47	1.18	0.57	0.34	0.72	0.501	0.367	0.780
*Acidaminococcaceae*	1.35	1.10	2.65	3.55	0.73	<0.001	0.643	0.088
*Nostocaceae*	0.29	1.25	<0.01	<0.01	0.60	0.197	0.254	0.999
*Xanthomonadaceae*	0.35	1.03	<0.01	<0.01	0.25	<0.001	0.013	0.969
*Porphyromonadaceae*	0.96	0.74	2.54	2.62	0.54	<0.001	0.717	0.893
*Pseudanabaenaceae*	0.60	0.87	0.07	0.08	0.45	0.011	0.458	0.970
*Erysipelotrichaceae*	0.45	0.76	1.23	1.42	0.33	0.033	0.514	0.694
*Bacillaceae*	1.19	0.03	0.08	0.03	0.48	0.244	0.089	0.944
*Bifidobacteriaceae*	0.34	0.21	0.99	0.29	0.36	0.289	0.786	0.146
*Eubacteriaceae*	0.64	0.54	2.42	2.58	0.37	<0.001	0.824	0.707
*Hyphomicrobiaceae*	0.29	0.29	0.26	0.43	0.12	0.592	0.973	0.299
*Bacteroidaceae*	0.25	0.11	0.27	0.24	0.08	0.326	0.229	0.816
*Flavobacteriaceae*	0.27	0.09	0.27	0.36	0.10	0.173	0.214	0.547
*Cytophagaceae*	0.27	0.19	1.63	1.14	0.67	0.012	0.900	0.446
*Spirochaetaceae*	0.07	0.02	0.70	0.25	0.17	0.005	0.789	0.041
*Oscillospiraceae*	0.09	0.06	0.52	0.34	0.09	<0.001	0.738	0.037
*Peptostreptococcaceae*	0.06	0.49	0.06	0.12	0.15	0.098	0.008	0.698
*Mycoplasmataceae*	0.13	0.07	0.35	0.15	0.13	0.132	0.623	0.139
*Spiroplasmataceae*	0.01	0.01	0.24	0.31	0.06	<0.001	0.928	0.368
*Caulobacteraceae*	0.43	0.08	<0.01	<0.01	0.14	0.006	0.016	0.968
*Rikenellaceae*	0.05	0.05	0.28	0.28	0.13	0.011	0.995	0.980
Others	1.79	1.88	4.13	1.94	0.72	0.100	0.921	0.016

^1^ Site: mucosa and feces. ^2^ F18^+^ *E. coli* challenge: no challenge: (−) and challenge: (+). ^3^ Mucosa vs. feces: effect of site on microbiota; M− vs. M+: effect of F18^+^ *E. coli* on jejunal mucosa-associated microbiota; F− vs. F+: effect of F18^+^ *E. coli* on fecal microbiota.

**Table 3:** Relative abundance of fecal and jejunal mucosa-associated microbiota at the genus level in pigs at d 21 after challenge with enterotoxigenic *E. coli* F18^+^.

Site ^1^	Mucosa		Feces			*p* Value ^3^		
Challenge ^2^	−	+	−	+	SEM	Site	M− vs. M+	F− vs. F+
*Prevotella*	22.15	15.01	33.78	30.10	3.60	<0.001	0.165	0.472
*Lactobacillus*	16.04	18.44	12.61	18.44	4.31	0.688	0.691	0.335
*Helicobacter*	16.83	32.83	0.51	0.65	5.20	<0.001	0.015	0.983
*Succinivibrio*	0.74	0.40	9.81	12.90	4.83	<0.001	0.930	0.420
*Campylobacter*	10.07	2.55	2.25	0.70	2.55	0.071	0.040	0.669
*Clostridium*	4.74	2.62	1.25	1.17	2.51	0.152	0.381	0.972
*Megasphaera*	1.90	1.27	6.16	4.44	1.68	0.002	0.703	0.297
*Mitsuokella*	2.75	1.38	2.99	4.91	1.40	0.102	0.397	0.240
*Pseudomonas*	3.53	6.57	<0.01	<0.01	1.74	<0.001	0.073	0.963
*Faecalibacterium*	1.58	1.34	4.70	4.08	0.97	<0.001	0.809	0.532
*Streptococcus*	3.49	4.76	1.45	1.92	2.34	0.135	0.579	0.839
*Phascolarctobacterium*	1.78	1.12	3.49	2.91	0.82	0.003	0.412	0.466
*Selenomonas*	0.35	0.33	4.39	2.51	1.15	<0.001	0.989	0.249
*Chlamydia*	4.12	2.44	<0.01	<0.01	2.07	0.056	0.515	0.967
*Dialister*	1.17	0.71	1.70	3.96	1.01	<0.001	0.518	0.002
*Roseburia*	0.54	0.49	2.03	1.94	0.40	<0.001	0.910	0.867
*Bifidobacterium*	0.24	0.42	2.23	0.46	0.85	0.220	0.883	0.133
*Acidaminococcus*	0.30	0.30	0.83	2.36	0.53	0.001	0.997	0.005
*Eubacterium*	0.33	0.38	1.34	0.94	0.44	0.041	0.922	0.454
*Oscillibacter*	<0.01	<0.01	1.68	0.54	0.48	<0.001	0.880	0.004
*Acinetobacter*	1.43	1.05	<0.01	0.02	0.59	0.024	0.619	0.922
*Gemmiger*	0.40	0.44	0.47	0.78	0.20	0.304	0.890	0.278
*Herbaspirillum*	<0.01	<0.01	1.08	0.69	0.39	<0.001	0.998	0.270
*Massilia*	0.52	1.05	<0.01	<0.01	0.50	0.051	0.850	0.984
*Bacillus*	1.44	0.02	<0.01	<0.01	0.57	0.219	0.084	0.999
*Coprococcus*	0.01	0.06	1.00	0.15	0.41	0.177	0.928	0.134
*Catenibacterium*	0.13	0.16	0.16	0.69	0.25	0.254	0.920	0.126
*Anaerovibrio*	0.09	0.22	0.44	0.27	0.15	0.115	0.434	0.339
*Dorea*	0.25	0.26	0.23	0.37	0.12	0.651	0.968	0.356
*Tepidimonas*	<0.01	<0.01	0.80	0.10	0.27	0.027	0.973	0.027
*Ruminococcus*	0.06	0.21	0.28	0.35	0.09	0.049	0.262	0.563
*Blautia*	0.16	0.50	<0.01	<0.01	0.15	0.004	0.039	0.879
*Brachyspira*	0.19	0.10	0.51	0.05	0.32	0.461	0.744	0.207
*Enterococcus*	0.60	0.01	<0.01	<0.01	0.29	0.293	0.147	0.999
*Treponema*	0.13	0.05	0.33	0.8	0.15	0.411	0.715	0.207
Others	1.74	1.77	1.12	1.54	0.39	0.225	0.812	0.445

^1^ Site: mucosa and feces. ^2^ F18^+^ *E. coli* challenge: no challenge: (−) and challenge: (+). ^3^ Mucosa vs. feces: effect of site on microbiota; M− vs. M+: effect of F18^+^ *E. coli* on jejunal mucosa-associated microbiota; F− vs. F+: effect of F18^+^ *E. coli* on fecal microbiota.

**Table 4:** Relative abundance of fecal and jejunal mucosa-associated microbiota at the species level in pigs at d 21 after challenge with enterotoxigenic *E. coli* F18^+^.

Site ^1^	Mucosa		Feces			*p* Value ^3^		
Challenge ^2^	−	+	−	+	SEM	Site	M− vs. M+	F− vs. F+
*Prevotella copri*	23.63	14.64	33.52	28.23	4.88	0.001	0.073	0.288
*Succinivibrio dextrinosolvens*	2.04	0.91	12.54	10.26	5.64	0.002	0.799	0.607
*Lactobacillus kitasatonis*	4.13	5.62	3.39	8.75	3.20	0.664	0.701	0.169
*Helicobacter mastomyrinus*	7.75	14.40	<0.01	<0.01	2.67	<0.001	0.079	0.999
*Helicobacter rappini*	5.23	15.59	0.90	1.07	3.96	0.001	0.011	0.965
*Faecalibacterium prausnitzii*	3.54	2.67	5.24	5.54	1.10	0.027	0.546	0.834
*Prevotella stercorea*	2.91	2.24	3.90	3.91	1.15	0.068	0.511	0.990
*Lactobacillus mucosae*	4.22	4.42	0.70	2.20	1.58	0.052	0.923	0.469
*Campylobacter coli*	9.03	0.43	1.11	0.27	2.65	0.143	0.021	0.824
*Phascolarctobacterium succinatutens*	2.65	1.45	3.92	3.43	0.97	0.024	0.232	0.622
*Lactobacillus delbrueckii*	1.55	1.86	3.14	2.60	1.23	0.265	0.837	0.715
*Mitsuokella jalaludinii*	2.40	1.05	2.14	3.70	1.05	0.253	0.288	0.358
*Dialister succinatiphilus*	1.93	1.19	2.35	5.12	1.29	0.005	0.484	0.010
*Roseburia faecis*	0.74	1.08	3.48	2.72	0.56	<0.001	0.674	0.346
*Streptococcus alactolyticus*	3.32	3.36	1.22	1.01	2.07	0.080	0.981	0.908
*Lactobacillus johnsonii*	1.03	1.37	1.64	1.54	0.90	0.516	0.693	0.908
*Chlamydia suis*	3.04	2.49	<0.01	<0.01	1.75	0.058	0.797	0.935
*Lactobacillus salivarius*	0.65	1.01	1.48	2.38	0.89	0.072	0.669	0.294
*Selenomonas lipolytica*	0.51	<0.01	2.96	0.85	0.87	0.015	0.621	0.036
*Gemmiger formicilis*	0.92	1.09	0.63	1.66	0.51	0.978	0.763	0.195
*Mitsuokella multacida*	1.62	0.69	0.31	1.30	0.54	0.522	0.226	0.199
*Megasphaera hominis*	0.50	0.30	2.62	0.89	1.09	0.130	0.877	0.172
*Acidaminococcus fermentans*	0.20	0.33	0.83	2.30	0.45	0.003	0.826	0.015
*Helicobacter equorum*	0.05	3.42	<0.01	0.04	1.21	0.163	0.054	0.980
*Selenomonas bovis*	0.25	0.08	2.17	0.90	0.60	0.004	0.827	0.235
*Prevotella* sp.	0.60	0.31	0.85	1.79	0.62	0.005	0.504	0.028
*Streptococcus hyointestinalis*	0.01	1.87	0.15	0.78	0.69	0.485	0.058	0.516
*Streptococcus infantarius*	1.11	1.72	0.30	0.27	0.89	0.062	0.478	0.974
*Acinetobacter radioresistens*	1.48	0.80	<0.01	<0.01	0.71	0.044	0.432	0.982
*Acinetobacter lwoffii*	1.99	0.42	<0.01	<0.01	0.54	0.013	0.027	0.991
*Campylobacter lanienae*	0.29	0.24	1.51	0.21	0.31	0.058	0.910	0.004
*Treponema porcinum*	0.13	0.08	1.48	0.31	0.37	0.016	0.915	0.011
*Campylobacter upsaliensis*	0.91	1.08	0.07	0.09	0.74	0.127	0.736	0.996
*Dorea longicatena*	0.51	0.60	0.26	0.45	0.17	0.247	0.723	0.428
Others	9.44	11.56	5.66	6.41	2.61	0.055	0.516	0.816

^1^ Site: mucosa and feces. ^2^ F18^+^ *E. coli* challenge: no challenge: (−) and challenge: (+). ^3^ Mucosa vs. feces: effect of site on microbiota; M− vs. M+: effect of F18^+^ *E. coli* on jejunal mucosa-associated microbiota; F− vs. F+: effect of F18^+^ *E. coli* on fecal microbiota.

**Table 5:** Immune and oxidative stress status in pigs after challenge with enterotoxigenic *E. coli* F18^+^.

Item	No challenge (A)	Challenge (B)	SEM	*p* value
Serum				
TNF-α, pg/mL	98.6	113.7	25.1	0.172
Protein carbonyl, nmol/mg of protein	1.74	2.10	0.45	0.396
Jejunal mucosa				
TNF-α, pg/mg of protein	1.15	1.31	0.27	0.551
Protein carbonyl, nmol/mg of protein	2.33	3.17	0.26	0.026

**Table 6:** Intestinal morphology and enterocyte proliferation in jejunum of pigs at d 21 post-challenge with enterotoxigenic *E. coli* F18^+^.

Item	No Challenge (A)	Challenge (B)	SEM	*p* Value
Jejunal mucosa				
Villus Height, µm	494	434	23	0.026
Crypt Depth, µm	209	224	30	0.169
VH:CD ^1^	2.60	2.14	0.30	0.044
Ki67^+^, %	51	55	8	0.632

^1^ Villus height to crypt depth ratio.

**Table 7:** Pearson correlation coefficients (r) between jejunal mucosa-associated microbiota and other variables measured in pigs after challenge with enterotoxigenic *E. coli* F18^+^.

Item ^1^	Family (r, *p* Value)	Species (r, *p* Value)
TNF-α, serum	*Lactobacillaceae* (0.30, 0.048)	*Succinivibrio dextrinosolvens* (−0.33, 0.030)
	*Veillonellaceae* (0.32, 0.034)	*Faecalibacterium prausnitzii* (−0.36, 0.017)
	*Enterobacteriaceae* (−0.32, 0.037)	*Lactobacillus mucosae* (0.51, <0.001)
	*Acidaminococcaceae* (0.36, 0.018)	*Megasphaera hominis* (0.49, 0.001)
	*Pseudomonadaceae* (−0.49, 0.001)	*Campylobacter lanienae* (0.49, 0.001)
	*Porphyromonadaceae* (0.43, 0.004)	
	*Eubacteriaceae* (0.33, 0.027)	
	*Cytophagaceae* (0.33, 0.027)	
	*Xanthomonadaceae* (−0.32, 0.036)	
	*Pseudanabaenaceae* (0.35, 0.018)	
	*Oscillospiraceae* (0.37, 0.015)	
	*Spiroplasmataceae* (0.33, 0.028)	
	*Caulobacteraceae* (−0.41, 0.005)	
TNF-α, mucosa	*Bifidobacteriaceae* (−0.63, <0.001)	*Mitsuokella jalaludinii* (0.45, 0.009)
		*Dialister succinatiphilus* (0.52, 0.002)
		*Mitsuokella multacida* (0.42, 0.016)
		*Acidaminococcus fermentans* (0.60, <0.001)
PC, serum	*Oscillospiraceae* (0.42, 0.005)	*Lactobacillus johnsonii* (0.49, 0.001)
	*Spiroplasmataceae* (0.69, <0.001)	*Megasphaera hominis* (0.45, 0.024)
		*Campylobacter lanienae* (0.66, <0.001)
PC, mucosa	*Erysipelotrichaceae* (0.32, 0.034)	*Acidaminococcus fermentans* (0.30, 0.044)
	*Bifidobacteriaceae* (−0.33, 0.031)	*Acinetobacter lwoffii* (0.33, 0.031)
	*Spiroplasmataceae* (0.44, 0.003)	
Villus height	*Veillonellaceae* (0.33, 0.031)	*Campylobacter coli* (0.33, 0.027)
	*Brachyspiraceae* (0.34, 0.023)	*Megasphaera hominis* (0.37, 0.013)
	*Pseudomonadaceae* (0.33, 0.026)	
	*Oscillospiraceae* (0.42, 0.041)	
Crypt depth	*Prevotellaceae* (−0.36, 0.019)	*Succinivibrio dextrinosolvens* (−0.50, 0.001)
	*Lactobacillaceae* (0.41, 0.006)	*Helicobacter rappini* (0.32, 0.039)
	*Succinivibrionaceae* (−0.39, 0.010)	*Faecalibacterium prausnitzii* (−0.55, <0.001)
	*Lachnospiraceae* (−0.40, 0.008)	*Dialister succinatiphilus* (0.30, 0.047)
	*Ruminococcaceae* (−0.36, 0.019)	*Roseburia faecis* (−0.38, 0.013)
	*Clostridiaceae* (−0.49, 0.001)	*Gemmiger formicilis* (−0.38, 0.013)
	*Pseudomonadaceae* (−0.30, 0.049)	*Megasphaera hominis* (0.41, 0.007)
	*Xanthomonadaceae* (−0.32, 0.034)	*Selenomonas bovis* (0.33, 0.035)
	*Pseudanabaenaceae* (0.36, 0.017)	*Campylobacter lanienae* (0.35, 0.020)
	*Mycoplasmataceae* (−0.34, 0.028)	*Dorea longicatena* (−0.40, 0.008)
VH:CD	*Lactobacillaceae* (−0.32, 0.034)	*Succinivibrio dextrinosolvens* (0.35, 0.021)
	*Lachnospiraceae* (0.31, 0.046)	*Helicobacter rappini* (−0.34, 0.024)
	*Clostridiaceae* (0.48, 0.001)	*Faecalibacterium prausnitzii* (0.43, 0.004)
	*Xanthomonadaceae* (0.32, 0.036)	*Roseburia faecis* (0.34, 0.024)
		*Acinetobacter lwoffii* (0.33, 0.032)
		*Dorea longicatena* (0.35, 0.020)

^1^ TNF-α: tumor necrosis alpha; PC: protein carbonyl; VH:CD: villus height to crypt depth ratio.

**Table 8:** Pearson correlation coefficients (r) between fecal microbiota and other variables measured in pigs after challenge with enterotoxigenic *E. coli* F18^+^.

Item ^1^	Family (r, *p* Value)	Species (r, *p* Value)
TNF-α, serum	*Veillonellaceae* (0.37, 0.013)	*Prevotella copri* (0.49, <0.001)
	*Succinivibrionaceae* (−0.33, 0.031)	*Succinivibrio dextrinosolvens* (−0.49, <0.001)
	Streptococcaceae (0.47, 0.001)	*Phascolarctobacterium succinatutens* (0.36, 0.016)
	*Acidaminococcaceae* (0.31, 0.041)	*Streptococcus alactolyticus* (0.36, 0.015)
	*Eubacteriaceae* (0.34, 0.025)	*Selenomonas lipolytica* (−0.30, 0.046)
	*Oxalobacteraceae* (−0.31, 0.044)	*Megasphaera hominis* (0.46, 0.002)
	*Cytophagaceae* (0.41, 0.006)	*Streptococcus hyointestinalis* (0.34, 0.024)
	*Flavobacteriaceae* (0.38, 0.010)	*Streptococcus infantarius* (0.37, 0.013)
		*Treponema porcinum* (−0.32, 0.037)
		*Dorea longicatena* (0.30, 0.049)
PC, serum	*Acidaminococcaceae* (0.31, 0.043)	*Prevotella copri* (0.35, 0.022)
Villus height	*Flavobacteriaceae* (0.33, 0.030)	*Dialister succinatiphilus* (0.41, 0.005)
		*Lactobacillus salivarius* (0.34, 0.025)
		*Mitsuokella multacida* (0.34, 0.022)
		*Treponema porcinum* (−0.30, 0.049)
Crypt depth	*Prevotellaceae* (0.45, 0.003)	*Prevotella copri* (0.47, 0.002)
	*Lactobacillaceae* (−0.34, 0.026)	*Succinivibrio dextrinosolvens* (−0.42, 0.004)
	*Succinivibrionaceae* (−0.42, 0.005)	*Lactobacillus kitasatonis* (−0.33, 0.030)
	*Ruminococcaceae* (0.35, 0.023)	*Faecalibacterium prausnitzii* (0.34, 0.023)
	*Acidaminococcaceae* (0.48, 0.001)	*Prevotella stercorea* (0.31, 0.040)
	*Oxalobacteraceae* (−0.33, 0.028)	*Dialister succinatiphilus* (0.42, 0.005)
	*Cytophagaceae* (0.34, 0.026)	*Lactobacillus johnsonii* (−0.37, 0.016)
	*Bifidobacteriaceae* (−0.32, 0.034)	*Selenomonas lipolytica* (−0.45, 0.003)
	*Flavobacteriaceae* (0.35, 0.020)	*Selenomonas bovis* (−0.41, 0.006)
	*Oscillospiraceae* (0.32, 0.034)	*Prevotella* sp. (0.45, 0.003)
VH:CD	*Prevotellaceae* (−032, 0.034)	*Selenomonas lipolytica* (0.39, 0.009)
	*Lactobacillaceae* (0.32, 0.036)	*Selenomonas bovis* (0.46, 0.002)
	*Succinivibrionaceae* (0.32, 0.038)	*Prevotella* sp. (−0.42, 0.005)
	*Ruminococcaceae* (−0.33, 0.028)	
	*Acidaminococcaceae* (−0.30, 0.049)	
	*Oscillospiraceae* (−0.31, 0.045)	
Ki67^+^	*Lactobacillaceae* (−0.42, 0.042)	*Lactobacillus mucosae* (−0.60, 0.002)
	*Ruminococcaceae* (0.48, 0.018)	*Lactobacillus delbrueckii* (−0.51, 0.011)
	*Oxalobacteraceae* (−0.52, 0.009)	*Phascolarctobacterium succinatutens* (0.41, 0.044)

^1^ TNF-α: tumor necrosis alpha; PC: protein carbonyl; VH:CD: villus height to crypt depth ratio; Ki67^+^: enterocyte proliferation rate in the crypts.

**Table 9:** Composition of basal diets (Exp. 1, 2, and 3; as-fed basis).

Item	Phase 1	Phase 2
Ingredient, %		
Corn, yellow dent	40.71	54.80
Soybean meal, 48% CP	22.00	23.50
Whey permeate	20.00	10.00
Blood plasma	6.00	3.00
Poultry meal	5.00	4.00
Poultry fat	3.50	1.80
L-Lys HCl	0.48	0.46
DL-Met	0.22	0.18
L-Thr	0.16	0.14
Dicalcium phosphate	0.25	0.68
Limestone	1.28	1.04
Vitamin premix ^1^	0.03	0.03
Mineral premix ^2^	0.15	0.15
Salt	0.22	0.22
Calculated composition:		
Dry matter, %	91.5	90.4
ME, kcal/kg	3488	3398
Crude protein, %	23.2	21.7
SID ^3^ Lys, %	1.50	1.35
SID Met + Cys, %	0.82	0.74
SID Trp, %	0.25	0.22
SID Thr, %	0.88	0.79
Ca, %	0.85	0.80
STTD ^4^ P, %	0.45	0.40
Total P, %	0.65	0.64

^1^ Vitamin premix: the vitamin premix provided the following per kilogram of complete diet: 6613.8 IU of vitamin A as vitamin A acetate, 992.0 IU of vitamin D3, 19.8 IU of vitamin E, 2.64 mg of vitamin K as menadione sodium bisulfate, 0.03 mg of vitamin B12, 4.63 mg of riboflavin, 18.52 mg of D-pantothenic acid as calcium pantothenate, 24.96 mg of niacin, and 0.07 mg of biotin. ^2^ Mineral premix: the trace mineral premix provided the following per kilogram of complete diet: 4.0 mg of Mn as manganous oxide, 165 mg of Fe as ferrous sulfate, 165 mg of Zn as zinc sulfate, 16.5 mg of Cu as copper sulfate, 0.30 mg of I as ethylenediamine di-hydroiodide, and 0.30 mg of Se as sodium selenite. ^3^ SID: standardized ileal digestible. ^4^ STTD: standardized total tract digestible.

## Data Availability

The data presented in this study are available upon request from the corresponding author.
